# Zinc Fingers Function Cooperatively with KRAB Domain for Nuclear Localization of KRAB-Containing Zinc Finger Proteins

**DOI:** 10.1371/journal.pone.0092155

**Published:** 2014-03-19

**Authors:** Wei Wang, Jinyang Cai, Yi Lin, Zikou Liu, Qihao Ren, Li Hu, Zan Huang, Mingxiong Guo, Wenxin Li

**Affiliations:** 1 State Key Laboratory of Virology, College of Life Sciences, Wuhan University, Wuhan, China; 2 Department of Biochemistry and Molecular Biology, College of Life Sciences, Wuhan University, Wuhan, China; University of South Florida College of Medicine, United States of America

## Abstract

Multiple nuclear localization domains have been identified in nuclear proteins, and they finely control nuclear import and functions of those proteins. ZNF268 is a typical KRAB-containing zinc finger protein (KRAB-ZFP), and previous studies have shown that the KRAB domain reinforces nuclear localization of KRAB-ZFPs by interacting with KAP1. In this study, we find that some of 24 zinc fingers of ZNF268 also possess nuclear localization activity. Results of mutagenesis studies suggest that KRAB and zinc fingers are both necessary, and they function both independently and cooperatively for the nuclear localization of ZNF268. However, the subnuclear targeting activities of KRAB and zinc fingers are different. KRAB targets proteins in nucleoplasm, but not in the nucleolus, which is mediated by interaction with KAP1, while zinc fingers target proteins in the whole nucleus uniformly. The cooperative activities of KAP1-KRAB-zinc fingers result in the precise nucleoplasmic, but not nucleolar localization of KRAB-ZFPs. Our studies reveal a novel mechanism for the subcellular localization of KRAB-ZFPs and may help us to further explore their biological functions.

## Introduction

KRAB-containing zinc finger proteins (KRAB-ZFPs) contain both the KRAB domain and some zinc fingers and represent the largest single family of transcriptional regulators in mammals [1], [2]. KRAB is found only in tetrapod vertebrates [3], [4] and functions as a transcriptional repressor domain with its corepressor KRAB associated protein 1 (KAP1) [1], [5]–[7]. The zinc finger domain in KRAB-ZFPs often consist of 10 or more tandem repeats of zinc fingers connected by a conserved stretch of seven amino acids (the H/C link) [1], [8]. KRAB-ZFPs regulate gene expression by binding target DNA sequence through the zinc finger domain, and the KRAB domain mediates the repression activity [1].

*ZNF268*, a typical KRAB-ZFP gene, was first isolated from a human embryo cDNA library [9]. Eight splice variants of the ZNF268 transcript, which are translated into ZNF268a and ZNF268b2 isoforms, have been detected [10]. ZNF268a contains KRAB and as many as 24 zinc fingers and may function as a transcriptional repressor [10], [11], whereas ZNF268b2 consists of only the 24 zinc fingers and has been demonstrated to be an IKK-associated protein participating in NF-κB-related pathways [12], [13]. ZNF268 expression is regulated by cAMP response element-binding protein 2 (CREB-2), which binds to the ZNF268 promoter localized within the first exon of the gene [14]. The function of ZNF268 has been implicated in human fetal liver development [15] and blood cell development [16]-[19]. A recent study of ours has demonstrated that aberrantly expressed ZNF268 may contribute to cervical carcinogenesis [12].

It has been reported that a variety of proteins possess multiple nuclear localization domains that may act cooperatively to increase nuclear accumulation more efficiently and allow fine control of nuclear import and function of the proteins [20]–[24]. We have previously observed that the KRAB domain is able to reinforce nuclear localization activity of KRAB-ZFPs by interacting with KAP1 [25]. Meanwhile, nuclear localization signal (NLS) of several zinc finger proteins have been identified that localize in the zinc fingers [26]–[29], consistent with the finding that NLS overlaps the DNA or RNA binding domains of nucleic acid-binding proteins [30].

In this study, another nuclear localization domain within the zinc fingers of ZNF268 was also identified. We found that both KRAB and zinc fingers were necessary for nuclear localization of the ZNF268a isoform. The two nuclear localization domains functioned cooperatively, though independently for the nuclear localization activity. The KRAB domain was found to target proteins in the nucleoplasm but was excluded from nucleoli, in contrast, the zinc fingers target proteins uniformly throughout the whole nucleus. We further demonstrated that interactions between KAP1, the corepressor of KRAB and zinc fingers determined the precise nucleoplasmic, but not nucleolar localization of KRAB-ZFPs.

## Materials and Methods

### Plasmid constructs

pEGFP-N1 (Clontech) was mutated at both the Kozak and the initial ATG codon (pEGFP-M1) to improve the expression of GFP fusion proteins and the accuracy of subcellular localization [31]. Full-length and truncated fragments of ZNF268a [a, a(1–4), a(1–8), a(1–12), a(1–16), a(1–20)], the nine regions [UD, KRAB, SD, ZF(1–4), ZF(5–8), ZF(9–12), ZF(13–16), ZF(17–20), ZF(21–24)] of ZNF268a and other mutants (UK, KS, KS4 and KS8) were amplified by PCR from pCMV-ZNF268a. Full-length and truncated fragments of ZNF268b2 [b2, b2(1–4) (or S4), b2(1–8) (or S8), b2(1–12), b2(1–16), b2(1–20)] were amplified from pCMV-ZNF268b2. The above fragments were digested and ligated into pEGFP-M1 to express GFP at the C terminus. KOX1 and ZNF300 genes were FLAG-tagged at the C terminus by ligation of the PCR fragments into pCMV-8tag-8 (Stratagene). PCR-directed mutagenesis were performed to generate the a(1–4)/mut, a(1–8)/mut, a(1–16)/mut and a/mut mutants with mutation at D8A/V9A or E16/17A-W18A in the KRAB domain with constructs a(1–4), a(1–8), a(1–16) and a-GFP as the template respectively and the corresponding primers. The primers for the above constructs are listed in Table S1.

### Cell culture and transfection

HeLa cells were cultured in Dulbecco's modified Eagle's medium (DMEM) supplemented with 10% (v/v) fetal bovine serum, penicillin, and streptomycin in a humidified 5% (v/v) CO_2_ incubator at 37°C. The day before transfection, cells were seeded on coverslips. Transfection of the plasmids was performed with Lipofectamine 2000 (Invitrogen) according to the manufacturer's instructions.

### Confocal microscopic analysis

Cultured cells were fixed with 4% (w/v) paraformaldehyde for 20 min at room temperature and permeabilized with 0.5% (v/v) Triton X-100 for 20 min at room temperature. Subsequently, the slides were incubated with primary antibody in buffers containing of 1% (w/v) BSA and 0.05% (v/v) Triton X-100 overnight, followed by incubation with tetramethyl rhodamine isothiocyanate (TRITC)- or fluorescein isothiocyanate (FITC)-conjugated secondary antibody (Pierce) for 1 h at 37°C. The slides were washed and the nuclei were stained with DAPI. The primary antibodies, anti-FLAG M2 and anti-KAP1, were purchased from Sigma-Aldrich and Cell Signaling Technology, respectively. For cells expressing GFP proteins, sample treatment was performed as described above without incubation with antibodies. Fluorescent images were taken with a BX61 microscope (Olympus) using the FV1000 configuration. In these images, the scale bars included represent 10 μm. Results of subcellular localization studies from three separate experiments are presented.

## Results

### KRAB and zinc fingers within ZNF268a contain distinct nuclear localization domains

**Figure 1:**
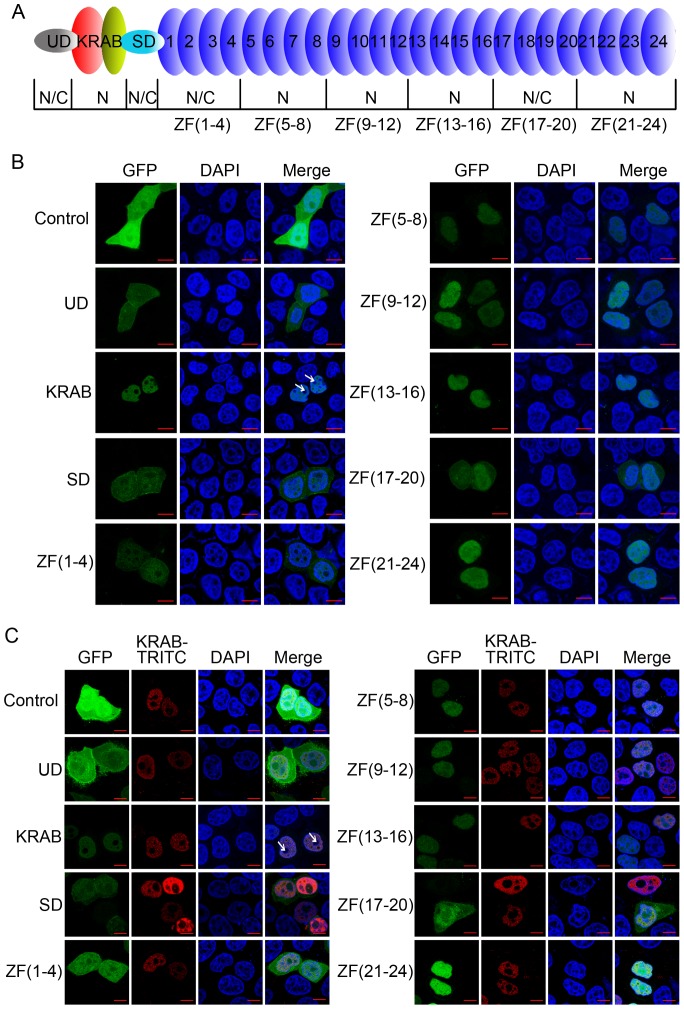
KRAB and zinc fingers of ZNF268 contain nuclear localization domains with different subnuclear localization. A, Schematic representation of ZNF268 structure. ZNF268 was divided into 9 regions. UD, unknown domain; SD, spacer domain; figures indicate each of the zinc fingers. The subcellular distribution of each region is indicated. N, nucleus; N/C, nucleus and cytoplasm; ZF, zinc fingers; numbers in parentheses near ZF indicate the numbers of zinc fingers the mutants contain. B, Subcellular localization of the nine regions of ZNF268. Each region of ZNF268 was fused to GFP and transfected into HeLa cells. Twenty-four hours later, confocal fluorescence analysis was performed; C, Different subnuclear distribution of zinc fingers and KRAB. Each region of ZNF268 fused to GFP was transfected into HeLa cells, together with FLAG-tagged ZNF268 KRAB. Twenty-four hours later, cells were subjected to confocal analysis. KRAB was labeled with TRITC. The white arrows indicate the nucleolus region with negative DAPI staining in the nucleus.

To delineate which region(s) possess nuclear localization activity, ZNF268a was divided into 9 segments and each was fused to GFP (Figure 1A) and their expressions were confirmed by western blot (Ref [25] and data not shown). As shown in Figure 1A–B, KRAB, ZF(5–8), ZF(9–12), ZF(13–16), and ZF(21–24) showed exclusive nuclear expression of GFP proteins, suggesting that the nuclear localization domains resided in the KRAB domain [25] and these zinc finger regions; proteins encoded by other constructs, e.g., UD, SD, ZF(1–4), and ZF(17–20), were present in both nucleus and cytoplasm, suggesting that these regions did not possess nuclear localization activity. Although both KRAB and some of the zinc fingers had nuclear localization activity, they showed different subnuclear distribution. KRAB localized in the nucleoplasm, but not nucleolus, as observed by absence of distribution in the area of DAPI-negative staining (Figure 1B) [32]; on the other hand, ZF(5–8), ZF(9–12), ZF(13–16), and ZF(21–24) were uniformly present throughout the whole nucleus (Figure 1B). This different subnuclear targeting activity between KRAB and the zinc fingers was further confirmed in colocalization assays by coexpression of KRAB and other regions of ZNF268a (Figure 1C).

### Zinc fingers and KRAB are both necessary for nuclear localization of ZNF268a

**Figure 2:**
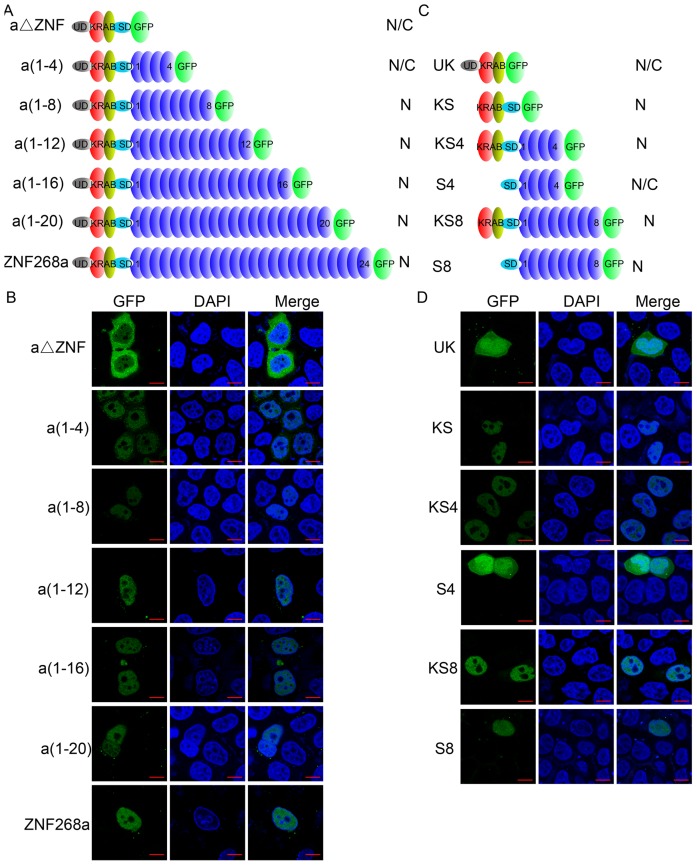
Both zinc fingers and KRAB domains are necessary for nuclear localization of ZNF268a. A and C, Schematic representation of ZNF268a and the truncation constructs. Numbers in parentheses indicate the number of zinc fingers the mutants contain. B and D, All the constructs were fused to GFP and transfected into HeLa cells and subjected to confocal analysis 24; N/C, nucleus and cytoplasm.

We examined which nuclear localization domains within KRAB and zinc fingers were necessary for nuclear localization of ZNF268a. A series of C-terminal deletion mutants of ZNF268a with different numbers of zinc fingers were fused to GFP (Figure 2A) and their expressions were confirmed by western blot (Ref [25] and data not shown). A construct lacking zinc fingers (aΔZNF) localized both to the nucleus and cytoplasm, and a construct with four zinc fingers [a(1–4)] showed nuclear accumulation of GFP with little GFP scattered within the cytoplasm (Figure 2A–B). As the number of zinc fingers increased, the mutants [a(1–8), a(1–12), a(1–16), and a(1–20)] and ZNF268a were exclusively localized in the nucleus (Figure 2A–B), suggesting these zinc fingers with nuclear localization activity were essential for nuclear localization of ZNF268a.

We recently demonstrated that the KRAB domain could reinforce nuclear localization of KRAB-ZFPs by interacting with KAP1 [25]. Though the aΔZNF construct contained KRAB, it didn't show nuclear accumulation, suggesting that other regions in the construct might counteract the nuclear localization activity of KRAB. To prove the hypothesis, additional mutants were generated (Figure 2C). KRAB with UD were present both in cytoplasm and nucleus, whereas KRAB with SD were exclusively in the nucleus. The results suggested that UD, rather than the SD region, suppressed the nuclear localization activity of KRAB. ZNF268a which also contains the UD domain is exclusively present in the nucleus (Figure 2A and 2B). The reason maybe that the combinational effects of UD, KRAB and zinc fingers determine its exclusive nucleus localization even though the UD conteracts the NLS activity of KRAB. To eliminate the possible counteracting activity of UD, we constructed four additional constructs deleted of the UD region (KS4, S4, KS8, and S8). SD and ZF(1–4) did not possess nuclear localization activity (Figure 1), and the fusion of the two (S4) was present both in nucleus and cytoplasm as expected. Addition of KRAB to S4 (KS4) was exclusively in the nucleus (Figure 2C–D), suggesting KRAB functions as nuclear localization activity in the context of zinc fingers. As KS8, S8 was also present in the nucleus, suggesting that the ZF (1–8) region possess nuclear localization activity (Figure 1) in the presence or absence of KRAB (Figure 2C and 2D). These results also suggest that KRAB and zinc fingers might function cooperatively as nuclear localization activity.

### KRAB and zinc fingers function cooperatively for nuclear localization of ZNF268a

**Figure 3:**
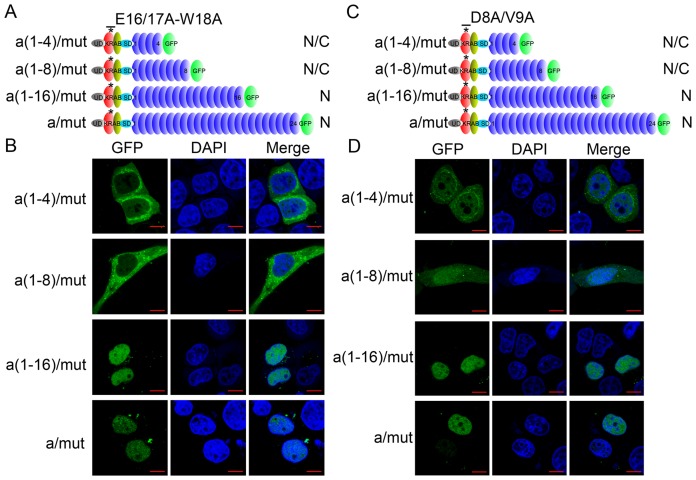
The effect of the KRAB mutation (E16/17A-W18A and D8A/V9A) on nuclear localization of ZNF268a and the truncated mutants. A, Schematic representation of a(1–4), a(1–8), a(1–16), and a-GFP mutations at the indicated site of the KRAB domain (E16/17A-W18A). B, The above constructs were transfected into HeLa cells for confocal analysis. The subcellular distribution of each construct is indicated in panel A. N, nucleus; N/C, nucleus and cytoplasm; C, Schematic representation of a(1–4), a(1–8), a(1–16), and a-GFP mutation at the indicated site of the KRAB domain (D8A/V9A); D, The above constructs were transfected into HeLa cells for confocal analysis.

Next, we examined the cooperative effect of KRAB and zinc fingers for nuclear localization of ZNF268a. Two functional blocks were identified, and their mutation decreased or abolished the nuclear localization activity of ZNF268 KRAB (D8A/V9A and E16/17A-W18A) [25]. Hence, we tested the subcellular localization of ZNF268a and its truncation mutants in the context of these KRAB mutations (Figures 3A, 3C). In the case of the E16/17A-W18A KRAB mutation, ZNF268a mutants with four fingers [a(1–4)/mut] showed more cytoplasmic distribution than wild-type protein (Figures 3B and 2B), and the a(1–8)/mut was present in the whole cells compared with the exclusively nuclear distribution of wild type (Figures 3B and 2B). These results further suggested that KRAB was functional as nuclear localization activity in the context of these zinc fingers. However, in the context of 16 [a(1–16)] or 24 zinc fingers (ZNF268a), proteins encoded by these mutants [a(1–16)/mut and a/mut] were all present in the nucleus to the same extent as the corresponding wild-type construct (Figures 3B and 2B). Similar results were also observed in the above constructs with the D8A/V9A KRAB mutation (Figure 3C and 3D). These results suggested that the increased zinc fingers function as nuclear localization activity when the nuclear localization activity of KRAB was decreased and KRAB and zinc fingers functioned cooperatively for nuclear localization of ZNF268a.

### KRAB and zinc fingers possess independently nuclear localization activity

**Figure 4:**
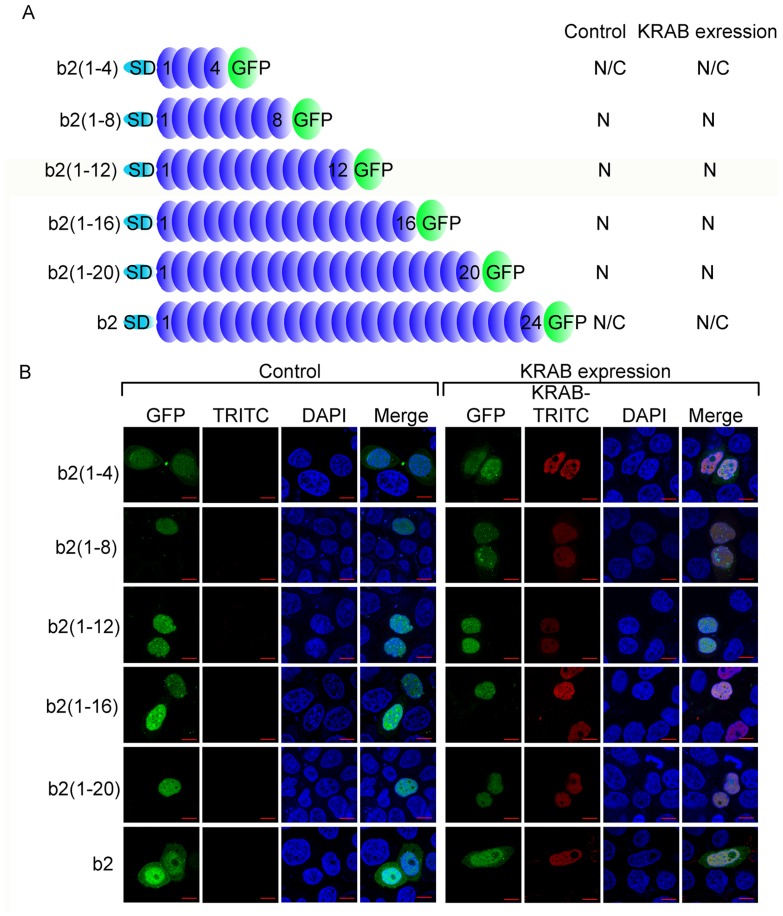
KRAB and zinc fingers contain two independent functional nuclear localization domains necessary for the subcellular localization of ZNF68. A, Schematic representation of ZNF268b2 and the truncation constructs. Numbers in parentheses indicate the number of zinc fingers the mutants contain. ZNF268b2 or the truncation mutants were fused to GFP and transfected into HeLa cells, together with control vector or FLAG-tagged ZNF268 KRAB. B, Twenty-four hours later, cells were subjected to confocal analysis. KRAB was labeled with TRITC. The subcellular localization pattern of each construct in control or KRAB expression group is indicated in panel A.

We tested whether the two types of nuclear localization domains (KRAB and zinc fingers) affect each other's nuclear localization activity. ZNF268b2 consisted of zinc fingers, which served as the model for us to study the effect of KRAB expression on the subcellular localization of zinc fingers (Figure 4A). As shown in Figure 4A–B, the ZNF268b2 truncation mutants with more than eight zinc fingers [b2(1–8), b2(1–12), b2(1–16), and b2(1–20)], except ZNF268b2, are exclusively in the nucleus, further confirming the zinc fingers possess nuclear localization activity. Additional KRAB expression did not alter the subcellular localization of the ZNF268b2 truncation mutants and ZNF268b2 (Figure 4). These results suggested that KRAB and zinc fingers are two independent nuclear localization domains, just as the respective repressor and DNA binding activities are independent functions for the regulation of transcription [1]. Our previous study [25] has demonstrated that the KRAB domain facilitates the nuclear import of KRAB-ZNF proteins. Though ZNF268b2 contains the full 24 zinc fingers that possess nuclear localization activity (Figure 1) and lacks UD domain with the counteracting effect (Figure 2), it didn't show exclusive localization in the nucleus (Figure 4). The reason maybe that the complicated spatial structure formed by these 24 zinc fingers in ZNF268b2 prevents its movement through the nuclear pore complex and this process needs the aid of the KRAB domain. Based on the reinforced nuclear localization activity by the KRAB domain as exemplified above by ZNF268b2, we would expect the same localization pattern of the ZNF268a mutants (D8A/V9A and E16/17A-W18A) with that of ZNF268b2 lacking KRAB domain. However, we still observed the exclusive nuclear localizations of these ZNF268a mutants (Figure 3). The reason maybe that the KRAB domain with mutations (D8A/V9A and E16/17A-W18A) may still retain some activities that facilitate these prolonged zinc fingers but not GFP alone [25] or the short zinc fingers (1–4) and (1–8) (Figure 3) to be imported into the nucleus such as facilitating the formation of the spatial structure necessary for entering through the nuclear pore complex.

### KRAB and zinc fingers cooperate for the precise nucleoplasmic, but not nucleolar, localization of KRAB-ZFPs

**Figure 5:**
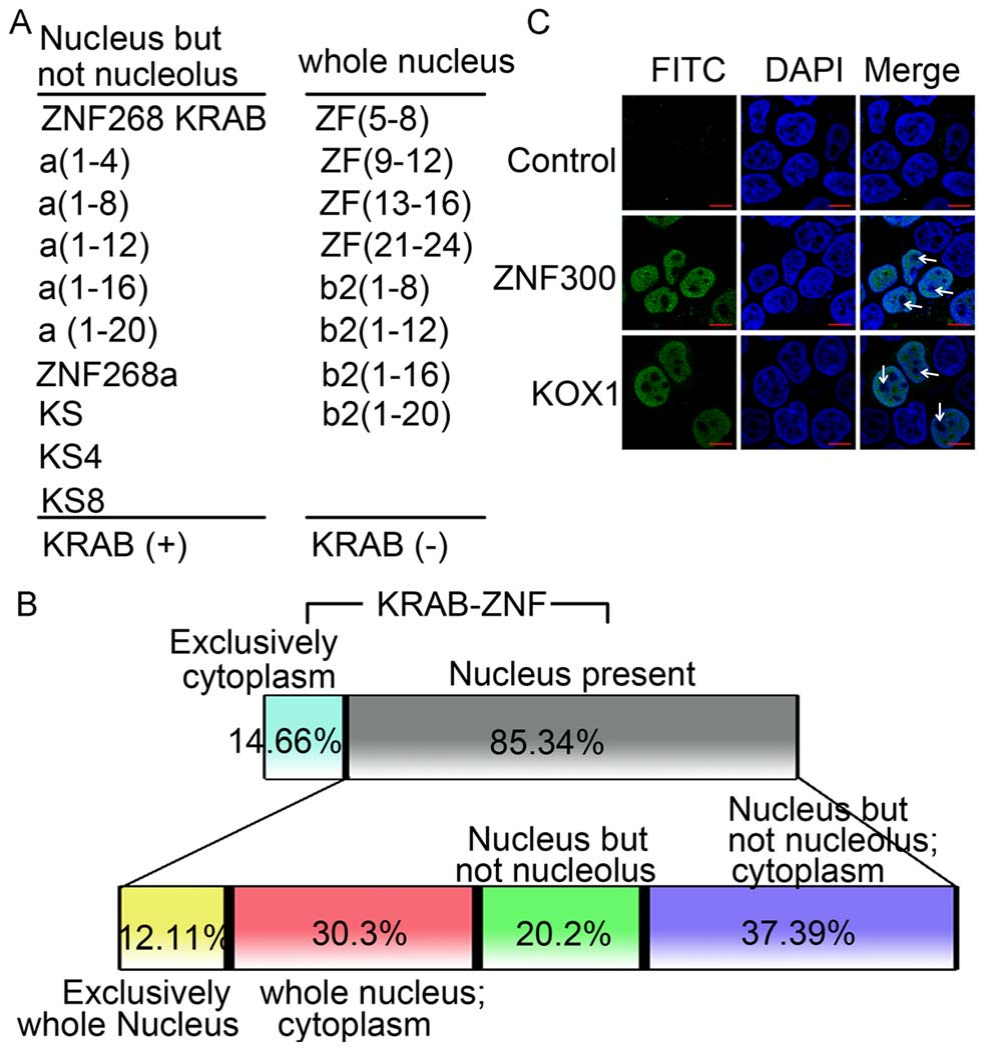
KRAB and zinc fingers function cooperatively for the precise nucleoplasmic, but not nucleolar, localization of KRAB-ZFPs. A, Analysis of subcellular localization of various ZNF268 mutants. B, Analysis of the endogenous subcellular localization of 116 human KRAB-ZFP genes. The subcellular localization data of 116 human KRAB-ZFP genes were retrieved from the Human Protein Atlas portal (www.proteinatlas.org), and the percentages of their subcellular localization were calculated based on the following rules. First of all, the localization pattern of these genes was analyzed based on their distribution in cytoplasm and nucleus. Then the genes that showed presence in the nucleus were further analyzed based on their distribution in cytoplasm and nucleolus. Specifically, they were classified into the following four groups: exclusively whole nucleus; whole nucleus and cytoplasm; nucleus but not nucleolus; nucleus but not nucleolus and cytoplasm. C, Subcellular localization of ZNF300 and KOX1 and their KRAB domains. KOX1 and ZNF300 genes were FLAG-tagged and transfected into HeLa cells. Control is the cells transfected with empty vector. The FLAG-tagged proteins were labeled with FITC. The white arrows indicate the nucleolus region with negative DAPI staining in the nucleus.

Individual nuclear localization domains in multiple NLS-containing proteins show differential properties [33], [34]. In this study, KRAB-containing proteins were present in the nucleoplasm but not in the nucleolus, whereas proteins without KRAB were distributed uniformly in the whole cell nucleus (Figure 5A), suggesting that the cooperative effects of KRAB and zinc fingers resulted in the precise localization of KRAB-ZFPs. To confirm this, data on the subcellular localization of 116 human KRAB-ZFP genes was retrieved from the Human Protein Atlas portal (www.proteinatlas.org) and subjected to analysis as described in Figure 5B legend. Consistent with their role as transcription factors, about 85% of the genes show expression in the nucleus. In the genes with nuclear expression, more than half (approximately 58%) showed nucleoplasmic (but not nucleolar) localization. In addition, the proportion of genes with exclusively nuclear (but not nucleolar) localization (20.2%) is greater than that of the exclusively whole nuclear proteins (12.11%) (Figure 5B). Furthermore, we overexpressed two typical KRAB-ZFP genes (ZNF300 and KOX1) in HeLa cells and found that they were both present in the nucleoplasm but not in the nucleolus, as expected (Figure 5C). Hence, KRAB and zinc fingers cooperate for the precise nucleoplasmic, but not nucleolar, localization of KRAB-ZFPs.

### Interaction between KRAB and KAP1 mediates the nucleoplasmic localization of KRAB-ZFPs

**Figure 6:**
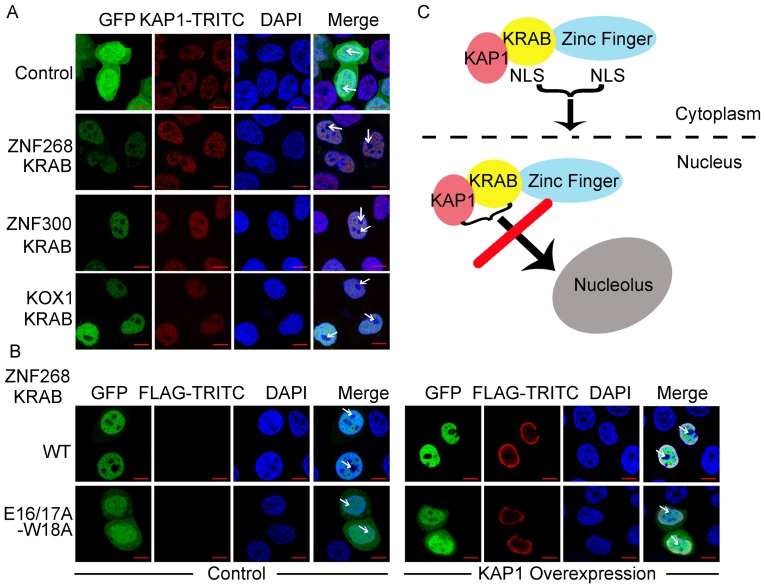
KRAB-KAP1 interaction mediates the nucleoplasmic localization of KRAB-ZFPs. A, Colocalization of KRAB and KAP1 in the nucleoplasm, but not nucleolus. HeLa cells were transfected with the indicated GFP expression plasmids. Twenty-four hours later, cells were incubated with anti-KAP1 antibody and subjected to labeling with TRITC conjugated secondary antibody for confocal analysis. B, The subcellular localization of ZNF268 KRAB mutants. Plasmids expressing wild-type (WT) or mutant ZNF268 KRAB-GFP (E16/17A-W18A) together with control (left panel) or FLAG-KAP overexpressing plasmid (right panel) were transfected into HeLa cells subjected to confocal analysis 24 hours later. The white arrows indicate the nucleolus region with negative DAPI staining in the nucleus. C, Model for subcellular localization of KRAB-ZFPs. The KRAB domain and Zinc finger domain both contain the NLS and their cooperative functions contribute to the precise nucleoplasmic, but not nucleolar localization.

Although nuclear localization domain was essential for nuclear import, targeting to the nucleolus has been shown to depend on interactions with nucleolar proteins, rRNA, and other nucleolar components [35]–[37]. We also observed the nuclear, but not nucleolar, localization of KRAB's corepressor protein KAP1, which colocalized with GFP-KRAB (ZNF268, KOX1, and ZNF300) fusion proteins (Figure 6A). KAP1 was able to block nucleolar sequestration of MDM2 by ARF [38]. The absence of nucleolar KRAB and KAP1 suggested that the interaction of these proteins may contribute to the inhibitory contact with nucleolar components, and thus they may be excluded from the nucleolus. To confirm this, we examined the subnuclear localization of the proteins encoded by ZNF268 KRAB mutant (E16/17A-W18A), which abolished the interaction with KAP1 and decreased the nuclear localization activity (Figure 6B) [25]. Careful analysis of the subnuclear localization revealed that the KRAB-GFP mutant (E16/17A-W18A) proteins were also present in the nucleolus, different from that of wild-type KRAB-GFP proteins as evidenced by the absent distribution in the nucleolus (Figure 6B). Furthermore, KAP1 overexpression was unable to rescue the nucleoplasmic, but not the nucleolar localization phenotype of the mutant (Figure 6B). Together, these results suggest that the interaction between KRAB and KAP1 may result in the precise nucleoplasmic, but not nucleolar, localization of KRAB-ZFPs (Figure 6C).

## Discussion

Multiple NLSs may act cooperatively for the nuclear import of proteins. Two types of nuclear localization domains (KRAB and zinc fingers) were identified within ZNF268 and function cooperatively in the following ways.

Firstly, neither KRAB nor zinc fingers alone are sufficient for nuclear localization of ZNF268a. This is supported by the following evidence: (i) loss of nuclear localization activity of KRAB by the D8A/V9A or E16/17A-W18A mutation does not alter the nuclear localization of a(1–20) and ZNF268a (Figure 3), suggesting that zinc fingers function as nuclear localization domain in this case, and KRAB alone is insufficient for nuclear localization of ZNF268a. KRAB may counteract the cytoplasmic localization effort of other regions, such as UD (Figure 2C–D) and function as nuclear localization activity in the context of zinc fingers, as observed in the a(1–4) and a(1–8) constructs (Figure 3); (ii) though ZNF268b2 consisting of 24 zinc fingers, it localizes in both cytoplasm and nucleus (Figure 4), compared to the exclusive nuclear localization of ZNF268a (Figure 2B) [25]. This suggests that zinc fingers need the aid of KRAB for nucleoplasmic localization of ZNF268a. Hence, this protein requires both the nuclear localization activities of the KRAB and zinc fingers to promote the nuclear translocation of ZNF268 proteins (Figure 6C).

Secondly, KRAB and zinc fingers function cooperatively for the precise nucleoplasmic, but not nucleolar, localization of KRAB-ZFPs. This is supported by the absence of nucleolar localization of KRAB-containing proteins (Figure 5A) and KRAB-ZFPs (Figure 5B), and other findings that no KRAB-ZFPs were detected in purified nucleoli [39]. We also demonstrated that KRAB-KAP1 interactions may contribute to the inhibitory contact with nucleolar components and thus be excluded from the nucleolus.

KRAB-ZFPs represent the single largest family of transcription regulators in mammals, however their functions remain largely unknown [2]. Our study may provide clues to investigate their biological functions. The cooperative NLS function of the KRAB domain and zinc fingers may facilitate the nuclear import and allow fine control of the function of KRAB-ZFPs. For example, our data show that ZNF268a isoform which contains the KRAB domain and zinc fingers is exclusively in the nucleus while ZNF268b2 isoform in both cytoplasm and nucleus. The two isoforms ZNF224 and ZNF255 of another gene also show the similar subcellular localization pattern to ZNF268 isforms [40]. This suggests that the combination effect of KRAB and zinc fingers may increase the efficiency of nuclear import. Meanwhile the different subcellular localization due to lack of KRAB may contribute to the different functions of these isoforms [12], [41]. Another interesting finding in our study is that the cooperative function of KRAB and zinc fingers contributes to their precise nuclear but not nucleolar localization. Their absences in nucleolus suggest that the transcriptional regulation by KRAB-ZFPs occur in the nucleus but not the nucleolus. Also, this subcellular feature also indicates that KRAB-ZFPs may not belong to a class of such repressors that are involved in construction of the nucleoli [42].

In conclusion, both zinc finger and KRAB domains contain nuclear localization activity and function cooperatively for the precise nucleoplasmic, but not nucleolar localization of KRAB-ZFPs, which may allow fine control of the biological functions. Further mechanistic and functional studies are clearly needed to dissect this fascinating future.

## Supporting Information

Table S1 **Primers designed for plasmid construction.**
(DOC)
