# Supplementary material for: Zinc Fingers Function Cooperatively with KRAB Domain for Nuclear Localization of KRAB-Containing Zinc Finger Proteins
Source: PLoS One. 2014 Mar 19;9(3):e92155. doi: 10.1371/journal.pone.0092155 (PMC3960175; doi:10.1371/journal.pone.0092155)
Supplement: Table S1 — Primers designed for plasmid construction. (DOC) [file pone.0092155.s001.doc]

Supplementary Table S1 Primers designed for plasmid construction

| Constructs | Primer name | Primer sequence |
| --- | --- | --- |
| ZNF268a-GFP | sense | 5' GGAAGATCTCCACCATGGCCACCAGGGTCCG 3' |
|  | antisense | 5' GCGTCGACTGATGTTTGTCATCTACAT 3' |
| a(1-4)-GFP | sense | 5' GGAAGATCTCCACCATGGCCACCAGGGTCCG 3' |
|  | antisense | 5' GCGTCGACTGCACATATGGTTTCTGTCCTGA 3' |
| a(1-8) -GFP | sense | 5' GGAAGATCTCCACCATGGCCACCAGGGTCCG 3' |
|  | antisense | 5' GCGTCGACTGTACATAAGGTTTCATT 3' |
| a(1-12) -GFP | sense | 5' GGAAGATCTCCACCATGGCCACCAGGGTCCG 3' |
|  | antisense | 5' GCGTCGACTGTTCAAATGGTTTCTCC 3' |
| a(1-16) -GFP | sense | 5' GGAAGATCTCCACCATGGCCACCAGGGTCCG 3' |
|  | antisense | 5' GCGTCGACTGCTCATGTGGTTTCTC 3' |
| a(1-20) -GFP | sense | 5' GGAAGATCTCCACCATGGCCACCAGGGTCCG 3' |
|  | antisense | 5' GCGTCGACTGTTTATAAGGGTTGACCC 3' |
| UD-GFP | sense | 5' GGAAGATCTCCACCATGGCCACCAGGGTCCG 3' |
|  | antisense | 5' GCGTCGACTGGGACTTGGTGATTTT 3' |
| SD-GFP | sense | 5' GGAAGATCTCCACCATGGATTGGCATCAGGA 3' |
|  | antisense | 5' GCGTCGACTGTCCAAAGGGTTTTTCGCC 3' |
| ZF(1-4) -GFP | sense | 5' GGAAGATCTCCACCATGTGCAGCTGTTGTGAGAAA 3' |
|  | antisense | 5' GCGTCGACTGCACATATGGTTTCTGTCCTGA 3' |
| ZF(5-8) -GFP | sense | 5' GGAAGATCTCCACCATGTGTAATGAATGTGGGAAAG 3' |
|  | antisense | 5' GCGTCGACTGTACATAAGGTTTCATT 3' |
| ZF(9-12) -GFP | sense | 5' GGAAGATCTCCACCATGTGCAATGAATGTGGCAAAGC 3' |
|  | antisense | 5' GCGTCGACTGTTCAAATGGTTTCTCC 3' |
| ZF(13-16) -GFP | sense | 5' GGAAGATCTCCACCATGTGTAGTGAGTGTCAGAAAG 3' |
|  | antisense | 5' GCGTCGACTGCTCATGTGGTTTCTC 3' |
| ZF(17-20) -GFP | sense | 5' GGAAGATCTCCACCATGTGCAGGGAATGCGGGAA 3' |
|  | antisense | 5' GCGTCGACTGTTTATAAGGGTTGACCC 3' |
| ZF(21-24) -GFP | sense | 5' GGAAGATCTCCACCATGTGCAGTCAATGTGAGAAAT 3' |
|  | antisense | 5' GCGTCGACTGATGTTTGTCATCTACAT 3' |
| UK-GFP | sense | 5' GGAAGATCTCCACCATGGCCACCAGGGTCCG 3' |
|  | antisense | 5' TTGGATCCCGTGGACAGGTCTGATTTG 3' |
| KS-GFP | sense | 5’ GGAAGATCTCCACCATGTGGGGACCTTTGT 3’ |
|  | antisense | 5’ GCGTCGACTGTCCAAAGGGTTTTTCGCC 3’ |
| KS4-GFP | sense | 5’ GGAAGATCTCCACCATGTGGGGACCTTTGT 3’ |
|  | antisense | 5’ GCGTCGACTGCACATATGGTTTCTGTCCTGA 3’ |
| KS8-GFP | sense | 5’ GGAAGATCTCCACCATGTGGGGACCTTTGT 3’ |
|  | antisense | 5’ GCGTCGACTGTACATAAGGTTTCATT 3’ |
| ZNF268b2-GFP | sense | 5’ GGAAGATCTCCACCATGGATTGGCATCAGGA 3’ |
|  | antisense | 5’ GCGTCGACTGATGTTTGTCATCTACAT 3’ |
| b2(1-4) –GFP | sense | 5’ GGAAGATCTCCACCATGGATTGGCATCAGGA 3’ |
|  | antisense | 5’ GCGTCGACTGCACATATGGTTTCTGTCCTGA 3’ |
| b2(1-8) –GFP | sense | 5’ GGAAGATCTCCACCATGGATTGGCATCAGGA 3’ |
|  | antisense | 5’ GCGTCGACTGTACATAAGGTTTCATT 3’ |
| b2(1-12) –GFP | sense | 5’ GGAAGATCTCCACCATGGATTGGCATCAGGA 3’ |
|  | antisense | 5’ GCGTCGACTGTTCAAATGGTTTCTCC 3’ |
| b2(1-16) –GFP | sense | 5’ GGAAGATCTCCACCATGGATTGGCATCAGGA 3’ |
|  | antisense | 5’ GCGTCGACTGCTCATGTGGTTTCTC 3’ |
| b2(1-20) –GFP | sense | 5’ GGAAGATCTCCACCATGGATTGGCATCAGGA 3’ |
|  | antisense | 5’ GCGTCGACTGTTTATAAGGGTTGACCC 3’ |
| ZNF268b2-GFP | sense | 5’ GGAAGATCTCCACCATGGATTGGCATCAGGA 3’ |
|  | antisense | 5’ GCGTCGACTGATGTTTGTCATCTACAT 3’ |
| ZNF268 KRAB-GFP | sense | 5’ TTCTCGAGCCACCATGGATTGGGGACCTTTGT 3’ |
|  | antisense | 5’ TTGGATCCCGTGGACAGGTCTGATTTG 3’ |
| ZNF300 KRAB-GFP | sense | 5’ TTCTCGAGCCACCATGGATCAGGGGTTAGTATCA 3’ |
|  | antisense | 5’ TTGGATCCCGTGGATAGATCCAATTTG 3’ |
| KOX1 KRAB-GFP | sense | 5’ TTCTCGAGCCACCATGGATGCTAAGTCACTAACT 3’ |
|  | antisense | 5’ TTGGATCCCGAGGATGGGTCTCTT 3’ |
| Flag ZNF268 KRAB | sense | 5’ TTGGATCCCCACCATGGATTGGGGACCTTTGT 3’ |
|  | antisense | 5’ TTCTCGAGTGGACAGGTCTGATTTG 3’ |
| ZNF300-Flag | sense | 5’ TTGGATCCCCACCATGGATCAGGGGTTAGTATCA 3’ |
|  | antisense | 5’ TTCTCGAGTTATGATTTTACCACTG 3’ |
| ZNF300 KRAB-Flag | sense | 5’ TTGGATCCCCACCATGGATCAGGGGTTAGTATCA 3’ |
|  | antisense | 5’ TTCTCGAGTGGATAGATCCAATTTG 3’ |
| KOX1-Flag | sense | 5’ TTGGATCCCCACCATGGATGCTAAGTCACTAACTGCCT 3’ |
|  | antisense | 5’ TTCTCGAGGTAAGCATTTTCTCTAATAT 3’ |
| KOX KRAB-Flag | sense | 5’ TTGGATCCCCACCATGGATGCTAAGTCACTAACTGCCT 3’ |
|  | antisense | 5’ TTCTCGAGAGGATGGGTCTCTT 3’ |
| KRAB (D8A/V9A) | sense | 5’ CCTTTGTCATTCATGGCCGCTTTTGTGGATTTTACC 3’ |
|  | antisense | 5’ GGTAAAATCCACAAAAGCGGCCATGAATGACAAAGG 3’ |
| KRAB (E16/17A-W18A) | sense | 5’ GTGGATTTTACCTGGGCCGCTGCCCAGCTGCTAGACCCA 3’ |
|  | antisense | 5’ TGGGTCTAGCAGCTGGGCAGCGGCCCAGGTAAAATCCAC 3’ |

For KRAB mutation (D8A/V9A and E16/17A-W18A), the underlined nucleotides indicated the alanine residues mutation. The other underlined nucleotides indicate the restriction enzyme sites.
